# A plastic surgeon's lament

**DOI:** 10.4103/0970-0358.53001

**Published:** 2009

**Authors:** Jimmy Mathew

**Affiliations:** Assistant Professor of Plastic Surgery, St. John's Medical College Hospital, Koramangala, Bangalore, India. E-mail: anujimy@gmail.com

O! Listen to the lament of a Plastic Surgeon,

Finishing a case, for me, is not urgent.

For I like to dwell…

Till everything is swell…

When other surgeons sleep I stay up and plan

That is the nature of the work of my clan.

When, snap! They start with the scalpel,

I leisurely fish out the pattern.

As a family, we are finicky as hell,

And drive all others up the wall.

The anaesthetists do not think we are so hot,

Making them grow roots, standing on a spot.

In training our residents we are very reluctant,

However much the poor chaps are persistent.

It is a pity that we stick with this mentality,

When other specialties take bites off our kitty.

Dentists, by droves, churned out every year,

Climb by the tooth roots to the face and ear.

Orthopods, muscle-bound as they are, as a band,

Clamour to be allowed to treat the hand.

During the eclipse, even earthworms wax poisonous,

As we see friendly dermatologists challenge us.

I would like to do a lot of cases, cosmetic,

And rake in the money, cool and ecstatic.

But am stuck with the penniless, with their parts cut off,

With lack of money and sleep, am very much put-off.

Brachial plexus, which nobody can really treat

It is our calling, with odds impossible to beat.

Raw areas galore, more than the occasional diabetic foot,

I have half a mind to chuck every thing, and scoot.

Numerous burns, though depressing, is life-saving

But earns less than a barber, doing cutting and shaving.

We exert ourselves to do what others can't

And end up doing what no one wants.

We still peer through the microscope

And see in the specialty great scope.

But God in his wisdom, has given us our perils

Who are we to complain, the poor devils?

He has entrusted us with poor, helpless patients

But has given us that elusive gem called ‘patience’

Do your Karma, without any expectation

And attain, by it, the ultimate emancipation.

